# Pain management with acupuncture in osteoarthritis: a systematic review and meta-analysis

**DOI:** 10.1186/1472-6882-14-312

**Published:** 2014-08-23

**Authors:** Taru Manyanga, Maria Froese, Ryan Zarychanski, Ahmed Abou-Setta, Carol Friesen, Michael Tennenhouse, Barbara L Shay

**Affiliations:** Department of Community Health Sciences, University of Manitoba, Winnipeg, Manitoba Canada; Surgery Rehabilitation Department, Seven Oaks General Hospital, Winnipeg, Manitoba Canada; Department of Internal Medicine, University of Manitoba, Winnipeg, Manitoba Canada; George & Fay Yee Center for Healthcare Innovation, University of Manitoba/Winnipeg Regional Health Authority, Winnipeg, Manitoba Canada; Neil John Maclean Health Sciences Library, University of Manitoba, Winnipeg, Manitoba Canada; Department of Physiotherapy, School of Medical Rehabilitation, University of Manitoba, Winnipeg, Manitoba Canada

**Keywords:** Acupuncture, Osteoarthritis, Pain, Functional mobility, Health-related quality of life, Systematic review, Meta-analysis

## Abstract

**Background:**

The utility of acupuncture in managing osteoarthritis symptoms is uncertain. Trial results are conflicting and previous systematic reviews may have overestimated the benefits of acupuncture.

**Methods:**

Two reviewers independently identified randomized controlled trials (up to May 2014) from multiple electronic sources (including PubMed/Medline, EMBASE, and CENTRAL) and reference lists of relevant articles, extracted data and assessed risk of bias (Cochrane’s Risk of Bias tool). Pooled data are expressed as mean differences (MD), with 95% confidence intervals (CI) (random-effects model).

**Results:**

We included 12 trials (1763 participants) comparing acupuncture to sham acupuncture, no treatment or usual care. We adjudicated most trials to be unclear (64%) or high (9%) risk of bias. Acupuncture use was associated with significant reductions in pain intensity (MD -0.29, 95% CI -0.55 to -0.02, *I*^*2*^ 0%, 10 trials, 1699 participants), functional mobility (standardized MD -0.34, 95% CI -0.55 to -0.14, *I*^*2*^ 70%, 9 trials, 1543 participants), health-related quality of life (standardized MD -0.36, 95% CI -0.58 to -0.14, *I*^*2*^ 50%, 3 trials, 958 participants). Subgroup analysis of pain intensity by intervention duration suggested greater pain intensity reduction with intervention periods greater than 4 weeks (MD -0.38, 95% CI -0.69 to -0.06, *I*^*2*^ 0%, 6 trials, 1239 participants).

**Conclusions:**

The use of acupuncture is associated with significant reductions in pain intensity, improvement in functional mobility and quality of life. While the differences are not as great as shown by other reviews, current evidence supports the use of acupuncture as an alternative for traditional analgesics in patients with osteoarthritis.

**Systematic review registration:**

CRD42013005405.

**Electronic supplementary material:**

The online version of this article (doi:10.1186/1472-6882-14-312) contains supplementary material, which is available to authorized users.

## Background

Osteoarthritis, the most common form of arthritis, is a progressive degenerative disease characterised by gradual loss of joint cartilage [1, 2], resulting in loss of movement and pain [3, 4]. It is the leading cause of disability among non-institutionalized adults [2], and is associated with major impacts on physical function and mobility [5]. Diagnosis is based on radiological changes, and clinical presentation of joint pain; including tenderness, limitation of movement, crepitus, joint effusion, and variable degrees of localized inflammation [5]. The prevalence, disability, and associated costs of treating osteoarthritis are expected to steadily increase over the next decades because of an aging population [6–8]. It is estimated that approximately 10% of men and 18% of women aged 60 years or older have symptomatic osteoarthritis worldwide [9, 10]. In the USA, job-related osteoarthritis costs up to $13 billion per year [9].

With no known cure [1], treatment of osteoarthritis is focused on symptom management. Pharmacological agents commonly prescribed include non-steroidal anti-inflammatory drugs (NSAIDs), acetaminophen, and in severe cases, narcotics [3, 5]. NSAIDs and acetaminophen are only marginally effective for short-term relief of osteoarthritic pain [2, 5, 11] and NSAIDs are associated with common adverse effects (e.g. upset stomach) [1, 5]. Analgesics are frequently prescribed in combination with other non-pharmacological therapies to decrease the dependency on analgesics [1, 2, 5]. These therapies include exercise [8, 12], weight reduction [2, 5, 8] and other complimentary/alternative therapies [2, 5, 13].

Acupuncture is reported to be effective in treating many conditions including, but not limited to, fibromyalgia [14] and chronic low back pain [15]; as well as chronic pain caused by osteoarthritis [16]. Due to its analgesic effects, acupuncture is widely used [2], cost effective [17, 18] and a relatively safe non-pharmacological treatment of musculoskeletal pain [1, 2, 19]. The ability of acupuncture to successfully manage osteoarthritic symptoms, either as monotherapy, or as an adjunct to usual medical care, remains uncertain [1, 20]. Inferences from previous systematic reviews that evaluated the effects of acupuncture on osteoarthritis have been speculative due to important limitations [2]. For example several previous reviews included trials in which electrical needle stimulation was performed [1, 2, 5] while another included data from non-randomized trials and quasi experiments [21].

The objective of the present systematic review was to identify, and synthesize data from prospective randomized controlled trials comparing acupuncture to sham acupuncture, usual care, or no treatment, in adults diagnosed with osteoarthritis.

## Methods

We conducted all aspects of this systematic review according to an *a priori* published protocol [22], and adhered to the Cochrane Handbook for Systematic Reviewers’ methodological guidelines. Our findings are reported in accordance to the Preferred Reporting Items for Systematic Reviews and Meta-Analyses [23]. The review question was formulated in consultation with an expert panel of clinicians and researchers with extensive knowledge synthesis experience, acupuncture and other therapeutic modalities.

### Populations, interventions, comparators, outcome measures and study designs (PICOS)

We posed the question, “What is the comparative efficacy and safety of acupuncture compared to sham acupuncture, usual care, or no treatment to reduce pain intensity in adults diagnosed with osteoarthritis?” (Additional file 1: Table S1). To address this question, we included randomized controlled trials of adults diagnosed with osteoarthritis (Additional file 1: Table S2). Our primary outcome measure was the reduction in pain intensity using a validated measurement tool. As secondary outcomes we compared functional mobility, health-related quality of life and procedural safety.

### Search strategy for identification of trials

We searched PubMed/MEDLINE (National Library of Medicine), EMBASE (Ovid), CENTRAL (the Cochrane Library), CINAHL (EbscoHost) and Natural Standard from inception to May 2014. We present the PubMed/MEDLINE strategy in Additional file 1: Table S3. To identify additional relevant citations, we conducted forward searches in Scopus and Web of Science. Our grey literature search included Osteoarthritis Research Society International (OARSI) conference proceedings (http://www.oarsi.org) from 2008 to 2014. To identify ongoing or planned trials, we searched the World Health Organization’s International Clinical Trials Registry Platform (ICTRP) and ClinicalTrials.gov. Finally, we hand-searched reference lists of narrative and systematic reviews and of the included trials for potentially relevant citation. We performed reference management in EndNote X6 (Thompson Reuters).

### Study selection

We used a two-step process for trial screening and selection. Two reviewers (TM and MF) independently screened the titles and abstracts to determine if a citation met the general inclusion criteria. We included randomized controlled trials (RCTs) of acupuncture administration to adults diagnosed with osteoarthritis. We excluded non-RCTs, trials involving animals and trials in which electro-needle stimulation was performed. Full details of inclusion and exclusion criteria are found in Additional file 1: Table S2. The full text of citations classified as *include* or *unclear* were reviewed independently with reference to the predetermined inclusion and exclusion criteria. Non-English full text citations were first translated and then reviewed independently. Disagreements between the two reviewers were resolved through consensus and by third-party adjudication, as needed.

### Data extraction and management

Two reviewers (TM and MF) independently extracted data from the included trial reports using standardized and piloted data extraction forms. Disagreements between the two reviewers were resolved by consensus or with adjudication of the content expert (BLS), as needed. The following data were extracted from each trial: patient demographics, interventions and comparators, trial outcomes, total acupuncture sessions, relevant co-interventions, length of each trial and duration of follow up.

### Assessment of methodological quality

We evaluated the internal validity of included trials using the Cochrane Risk of Bias tool [24]. This tool consists of six domains (sequence generation, allocation concealment, blinding, incomplete outcome data, selective outcome reporting, and ‘other’ sources of bias). Each separate domain was rated having a ‘low’, ‘unclear’ or ‘high’ risk of bias. If one or more individual domains were assessed as having a high risk of bias, the overall assessment was rated as having a high risk of bias. The overall risk of bias was considered low only if all components were rated as having a low risk of bias. The risk of bias for all other studies was rated as unclear.

### Adequacy of acupuncture

Due to significant variability and lack of standardization, we decided, *a priori*, to consider acupuncture therapy regardless of frequency of administration, duration of each session, number, depth of penetration and location of needles, as well as the designation of the acupuncturist. In most trials, acupuncturists ensured *de chi* was achieved and performed manual needle stimulation at least once during each session. The most commonly used acupuncture points were ST34, ST36, Xiyan, GB34 and SP9 (Additional file 1: Table S5).

### Measures of treatment effect

We analyzed all outcomes using Review Manager (RevMan, version 5.2) (24). Pooled continuous data were expressed as mean difference (MD) with 95% confidence intervals (CI). We calculated standardized mean differences (SMD) when multiple scales were used to measure the same outcome in different trials. Pooled dichotomous data are presented as odds ratios (OR). We used the random effects model for all analyses and quantified statistical heterogeneity using the *I*^*2*^ statistic. If significant heterogeneity was detected (*I*^*2*^ > 50%), sensitivity analyses were conducted to identify the source (s) of the heterogeneity. We assessed publication bias by viewing the overlap of confidence intervals and using funnel plot techniques [25].

### Measurement tools

Pain intensity was measured using the visual Analogue scale. Two variations of this scale (0–10 cm or 0–100 mm) [26] were used in the included trials. To assess functional mobility, the Western Ontario and McMaster Universities Osteoarthritis Index (WOMAC) scale was used [27]. This scale rates activities according to degree of difficulty (0 = none and 4 = extreme difficult). Other trials used the Knee injury and Osteoarthritis Outcome Score (KOOS) with five categories (0 = none and 5 = extreme) difficulty) [28].

### Subgroup/sensitivity analysis

For the primary outcome of pain intensity, we performed the following *a priori* subgroup analyses: Clinical considerations: We hypothesized greater reduction in pain intensity when 1) acupuncture was compared to sham acupuncture, usual care or no treatment or; 2) trials had ten or more acupuncture sessions or 3) had intervention periods longer than 4 weeks of intervention. Methodological considerations: We hypothesised greater effect sizes among 4) trials with unclear or high risk of bias; or 5) single centre trials.

## Results

**Figure 1:**
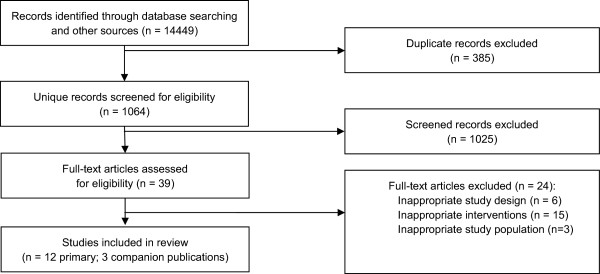
**Study Flow diagram.**

Of the 14449 citations identified through electronic and hand searches, we included 12 unique trials enrolling a total of 1763 participants [16, 29–39] (Figure 1). Trials were published between 1989 and 2013; 75% were single-centre trials. Nine trials [13, 29–34, 36, 39] were conducted in physiotherapy outpatients departments while three [35, 37, 38] occurred in primary care centres. All trials were published in English language journals. Nine trials were conducted in Europe [13, 30–32, 35–39], two in Iran [29, 34] and one in Israel [33]. Manual needle stimulation was performed in most (75%) of the trials. Only three trials [30, 36, 39] did not perform manual needle stimulation.

**Figure 2:**
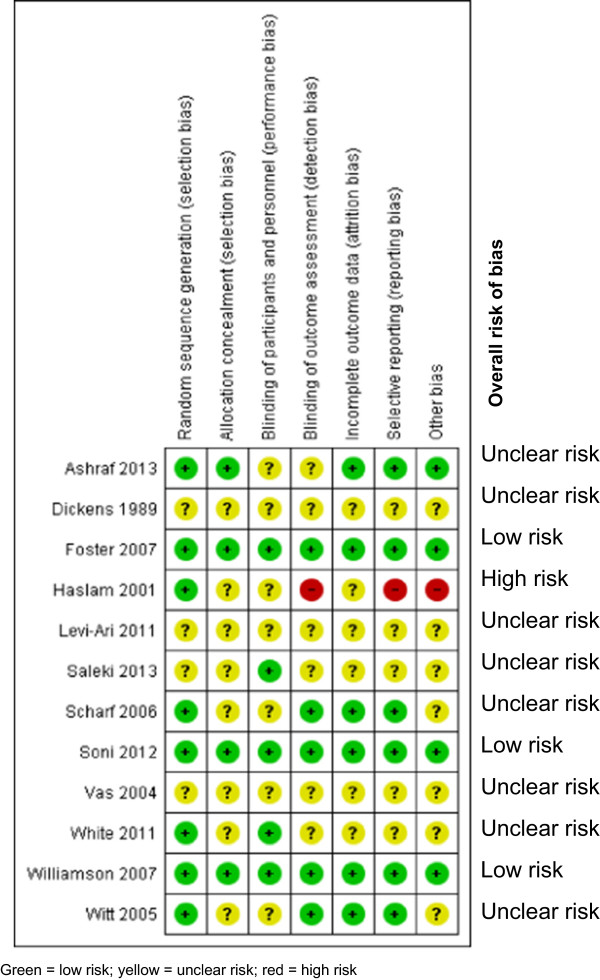
**Summary risk of bias assessment.**

Duration of interventions ranged from two to twelve weeks, with total follow-up durations ranging from four to 52 weeks. The age of participants ranged from 39 to 72 years; 65% of trial participants were female (Table 1). Eight trials (67%) were adjudicated to be unclear risk of bias [13, 29, 30, 33–35, 37, 38], three (25%) were considered low risk [31, 36, 39] and one trial (8%) was classified as high risk of bias [32] (Figure 2). Four trials compared true acupuncture to a sham acupuncture [31, 33, 37, 38] six trials used ‘usual care’ as the control [29, 32, 34–36, 39], one [13] used a waiting list control (i.e. no treatment), and one trial [30] used mock transcutaneous electrical nerve stimulation in the control group. From a practical perspective, we considered conservative therapy, pharmacological treatments, and exercises as ‘usual care’.

**Table 1:** **Characteristics of Included studies**

Study	RCT type	Number of participants (Acupuncture/control)	Setting	Mean age (SD)	Control intervention	Total acupuncture sessions	Co-interventions	Duration of intervention	Duration of follow up
**Scharf,** **2006** [35]	Multicentre	691 (326/365)	Primary care centres	62.90 (7.36)	Usual care (analgesics and NSAIDs; physio sessions)	10	NSAIDs	6 weeks	26 weeks
**Foster,** **2007** [31]	Multicentre	233 (117/116)	Outpatients physio departments	62.96 (10.45)	Sham acupuncture	6	NSAIDs and analgesics	3 weeks	52 weeks
**Witt,** **2006** [13]	Multicentre	219 (149/70)	Outpatients physio departments	64 (7)	Waiting list	12	NSAIDs	8 weeks	52 weeks
**White,** **2011** [38]	Single centre	147 (74/73)	Primary care centers	66.75 ( 8.29)	Sham acupuncture	8	NR	4 weeks	NR
**Williamson,** **2007** [39]	Single centre	121 (60/61)	Outpatients physio department	71.36 (8.22)	Usual care (supervised exercises; advice leaflet)	6	NR	6 weeks	12 weeks
**Vas,** **2004** [37]	Single centre	97 (48/49)	Primary care centre	65.70 (15.76)	Sham acupuncture	11	NSAIDs	12 weeks	NR
**Soni,** **2012** [36]	Single centre	56 (28/28)	Outpatients physio department	68.75 (8.7)	Usual care (exercises and advice leaflet)	6	NR	8 weeks	12 weeks
**Levi**-**Ari,** **2011** [33]	Single centre	55 (28/27)	Outpatients physio department	71.7 (8.6)	Sham acupuncture	16	NSAIDs	8 weeks	12 weeks
**Saleki,** **2013** [34]	Single centre	40 (20/20)	Outpatients physio departments	NR	Usual care (physio sessions; hot packs)	12	NR	4 weeks	NR
**Haslam,** **2001** [32]	Single centre	32 (16/16)	Outpatients physio department	39-77 (range)	Usual care (supervised demonstration of exercise; advice leaflet)	6	NR	6 weeks	14 weeks
**Ashraf,** **2013** [29]	Single centre	40 (20/20)	Outpatients physio department	55.2 (7.15)	Usual care (in-shoe wedge; follow up phone calls)	10	NR	3 weeks	NR
**Dickens,** **1989** [30]	Single centre	12 (7/5)	Outpatients physio department	48-77 (range)	Mock TENS	6	NR	2 Weeks	4 Weeks

*Physio* = physiotherapy; *NR* = not reported; *SD* = standard deviation; *TENS* = transcutaneous electrical nerve stimulation; *NSAIDs* = non-steroidal anti-inflammatory drugs.

### Primary outcome: pain intensity

**Figure 3:**
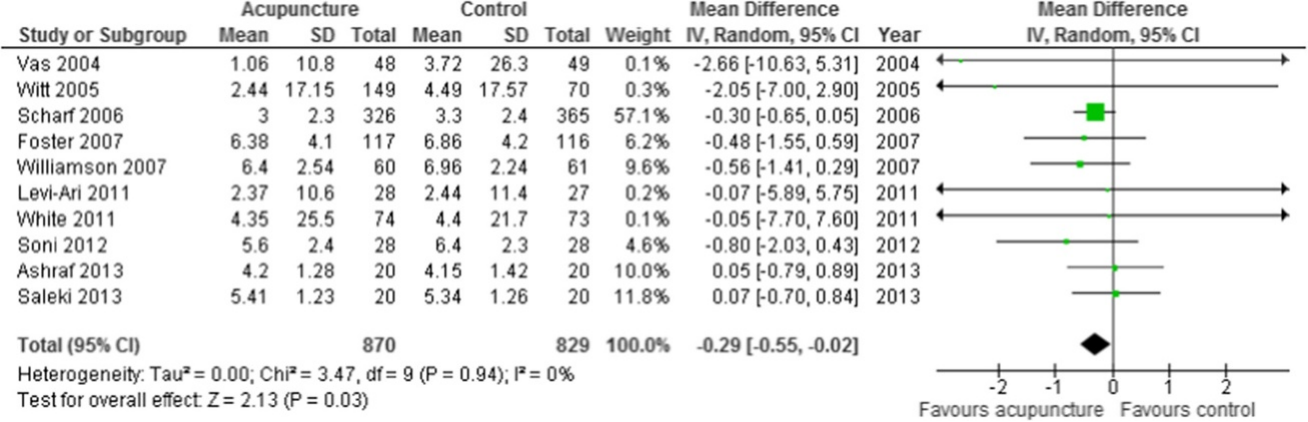
**Pain intensity (Visual Analog Scale).**

Ten trials [13, 29, 31, 33–39] involving 1699 participants contributed pain intensity data for meta-analysis (Figure 3). Overall, the use of acupuncture in adults with osteoarthritis was associated with significantly reduced osteoarthritic pain on the visual analogue scale (MD -0.29, 95% CI -0.55 to -0.02, *I*^*2*^ 0%). Publication bias could not be excluded (Additional file 1: Figure S1) due to the modest number of included trials [40]. We evaluated the efficacy of acupuncture for osteoarthritic pain according to predefined subgroups. Compared to intervention durations of ≤ 4 weeks, longer intervention periods were associated with significant difference reductions in pain intensity (MD -0.38, 95% CI -0.69 to -0.06, *I*^*2*^ 0%, 6 trials, 1239 participants) (Additional file 1: Figure S2).

We observed no significant differences in pain intensity according to type of comparator (sham acupuncture vs. other treatments) and use of co-interventions (NSAIDs vs. none). In a subgroup analysis of trials at low risk of bias, the pooled mean difference for pain intensity associated with acupuncture was -0.59 (95% CI -1.18 to -0.00, *I*^*2*^ 0%, 3 trials, 410 participants) (Additional file 1: Figure S3). Pain intensity was not significantly different in the subgroups of single centre or multicentre trials, nor did it differ between trials with adequate vs. unclear blinding of participants and assessors.

### Secondary outcomes: functional mobility and HRQoL

**Figure 4:**
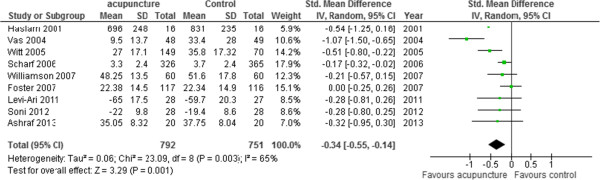
**Functional mobility (multiple scales).**

**Figure 5:**
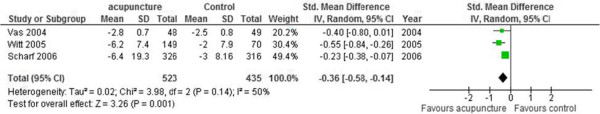
**Health-related quality of life (multiple scales).**

Nine trials [13, 29, 31–33, 35–37, 39] involving 1543 participants contributed functional mobility data (Figure 4). Functional mobility assessed at the end of trials was significantly improved in the acupuncture groups compared to control groups (SMD -0.34, 95% CI -0.55 to -0.14), but there was moderate statistical heterogeneity (I^2^ 65%) between the results of the included trials. We explored this heterogeneity by excluding the trial with the longest study intervention (12 weeks) as it had demonstrated the greatest benefit [37]. The results of this outlying trial were statistically different from the other trials (*I*^*2*^ 93%). With this trial excluded, statistical heterogeneity was reduced (*I*^*2*^ 15%), and acupuncture remained associated with improvements in functional mobility. Three trials [13, 35, 37] involving 958 participants reported HRQoL. Acupuncture was associated with significant improvements in HRQoL at the end of intervention period (SMD -0.36, 95% CI -0.58 to -0.14, *I*^*2*^ 50%) (Figure 5).

### Adverse events

pt?>Two [13, 35] of 11 trials involving 861 participants systematically reported adverse events (Additional file 1: Figure S4). The odds ratio for any adverse event associated with acupuncture compared with the controls was 1.44, (95% CI 0.77 to 2.71, *I*^*2*^ 39%). With regard to the remaining trials, one trial reported five adverse events in the acupuncture group (pain, sleepiness, fainting, nausea, and localized swelling) but omitted to report adverse events the control group [31]. A second trial reported bruising associated with acupuncture sites, while a third trial [38] presented a table of side effects without incidence rates in either group [37].

## Discussion

In this systematic review, we found acupuncture administered to adults with osteoarthritis to be associated with a statistically significant reduction in pain intensity, improved functional mobility and improved health-related quality of life. Reductions in pain were greater in trials with longer intervention periods. Though under-reported and inconsistently described, major adverse events with acupuncture were not reported. Subgroup analyses suggest that acupuncture is most effective for reducing osteoarthritic pain when administered for more than four weeks. Outcome assessment for the majority of trials occurred immediately following the intervention period and thus the durability of treatment effects are unknown.

Given the chronic nature of osteoarthritic pain, the presence of inflammation and well-established nociceptive pathways may necessitate a threshold dose or duration of treatment prior to clinical effect [41, 42]. As such, guidelines recommend on average, 10 acupuncture treatment sessions for chronic conditions [41, 43]. This recommendation is supported by pathophysiologic and anatomic studies showing how sustained nociceptive input caused by osteoarthritis can have profound effects on the central nervous system causing pathologic neuroplastic changes [41]. The controlled stimulation of peripheral nociceptors with acupuncture may reverse such pathologic neuroplasticity in the central nervous system; especially when administered over a prolonged period [41]. Optimal dose density, (i.e. sessions per week and duration of each session), remains to be established.

Although our review demonstrated statistically significant reductions in pain intensity and improvements in both functional ability and quality of life, the clinical relevance of these findings is of great importance. Pooled treatment effects observed in our review did not meet previously established thresholds (effect size of 0.39 and 0.37 for pain and function respectively) for the minimal clinically important difference (MCID) in patients with osteoarthritis [44]. Determination of what constitutes MCID in osteoarthritic patients is however subject to considerable debate due in part to the use of ‘intuitive sense’ [44, 45]. Lack of consensus, as evidenced by three different recommendations [5, 44–46], makes it difficult to conclude that our findings are clinically irrelevant and not merely an issue of “judgement”. Further investigations are needed to establish a relevant definition of MCID for therapeutic interventions of osteoarthritis.

Results from previous reviews [1, 2, 5, 47] of acupuncture conducted in participants with osteoarthritis are inconsistent. Two reviews [2, 5] found only short term reductions in pain and improvement of function, while another [47] found both short and long term benefits for acupuncture. One previous review [1] concluded reduced pain but no change in function. Some of these reviews included a subset of the trials included in this review and/or analyzed data from trials with substantial variability in the definition and application of acupuncture. Inconsistent findings may also relate to the inclusion of trials comparing traditional acupuncture to minimal/superficial acupuncture as well as trials studying electro-acupuncture. Electrical needle stimulation can enhance the effects of acupuncture [19, 45], and thus equating electro-acupuncture to traditional acupuncture is not an accurate representation of the efficacy of acupuncture. Previous inclusion of non-randomized trials or quasi-experiments may have also exaggerated effect estimate [21]. In our systematic review we excluded non randomized trials, trials in which superficially penetrating needles were used as sham acupuncture, and trials where electrical needle stimulation was performed in the treatment arm.

### Strengths

The strengths of our review included completeness of our search strategy, including searching multiple bibliographic databases, trial registries and conference proceedings for randomized controlled trials comparing traditional acupuncture to a ‘true control’ (e.g. sham/placebo acupuncture, exercise, or waiting list). Furthermore, we focused on patient-centered outcomes, and evaluated the efficacy of acupuncture in the context of its safety profile. Finally, we used an *a priori* published protocol, and followed established methodological guidelines for synthesizing the evidence.

### Weaknesses

Our review may be limited by methodological challenges inherent in the included trials. From the included trials, 75% were adjudicated to be of unclear or high risk of bias. We decided, *a priori*, to consider acupuncture therapy regardless of frequency of administration, duration of each session, number and location of needles, as well as the designation of the acupuncturist. While these variables may affect the adequacy of acupuncture administered, we’ve also acknowledged a lack of consensus on what defines ‘usual care’ in acupuncture. It is unknown if this *a priori* methodological decision represents a source of systemic error or natural variability representative of current practice. Most trials included in our review provided inadequate descriptions of blinding procedures or methods to ensure allocation concealment. Failure to maintain allocation concealment or blinding in trials has been associated with inflated effect estimates [2, 5, 15, 46, 47].

## Conclusions

The use of acupuncture is associated with significant reductions in pain intensity, improvement in functional mobility and quality of life. While the differences are not as great as shown by other reviews, current evidence supports the use of acupuncture as an alternative for traditional analgesics in patients with osteoarthritis.

### Ethical approval

No ethics approval was sought since this study involved a synthesis and analysis of data from previously published research.

### Data access

TM had full access to all of the data in this review and takes responsibility for the integrity of the data and the accuracy of its analysis.

## Electronic supplementary material

Additional file 1: **Table S1.** Research question using PICOS structure. **Table S2.** Study eligibility criteria. **Table S3.** PubMed/MEDLINE search strategy. **Table S4.** Needle location for each trial. **Figure S1.** Funnel plot for pain intensity. **Figure S2.** Subgroup analysis (clinical considerations). **Figure S3.** Subgroup analysis (methodological considerations). **Figure S4.** Adverse Events. (DOCX 520 KB)

## Abbreviations

MD: Mean Difference
SMD: Standardized Mean Difference
CI: Confidence Interval
SE: Standard Error
NSAIDs: Non-steroidal anti-inflammatory drugs
PICOs: Populations, Interventions, Comparators, Outcomes, Study designs
OARSI: Osteoarthritis Research Society International
OR: Odds Ratio
HRQoL: Health Related Quality of Life
MCID: Minimal Clinically Important Difference
RCT: Randomized Controlled Trials
DF: Degrees of Freedom
IV: Inverse Variance
Chi^2^: Chi-squared
I^2^: I-squared
M-H: Mantel-Haenszel
Tau^2^: Tau-squared
VAS: Visual Analogue Scale
WOMAC: Western Ontario and McMaster Universities Osteoarthritis Index
KOOS: Knee injury and Osteoarthritis Outcome Score.
