# Supplementary material for: Pain management with acupuncture in osteoarthritis: a systematic review and meta-analysis
Source: BMC Complement Altern Med. 2014 Aug 23;14:312. doi: 10.1186/1472-6882-14-312 (PMC4158087; doi:10.1186/1472-6882-14-312)
Supplement: Supplementary file 1 — Additional file 1: Table S1. Research question using PICOS structure. Table S2. Study eligibility criteria. Table S3. PubMed/MEDLINE search strategy. Table S4. Needle location for each trial. Figure S1. Funnel plot for pain intensity. Figure S2. Subgroup analysis (clinical considerations). Figure S3. Subgroup analysis (methodological considerations). Figure S4. Adverse Events. (DOCX 520 KB) [file 12906_2013_1895_MOESM1_ESM.docx]

**Supplementary Data (Web Only Files)**

Taru Manyanga, Maria Froese, Ryan Zarychanski, Ahmed M. Abou-Setta, Carol Friesen, Michael Tennenhouse, and Barbara L. Shay. **Pain management with acupuncture in osteoarthritis: A systematic review and meta-analysis**

Table S1. Research question using PICOS structure

Table S2. Study eligibility criteria

Table S3. PubMed/MEDLINE search strategy

Table S4. Needle location for each trial

Figure S1. Funnel plot for pain intensity

Figure S2. Subgroup analysis (clinical considerations)

Figure S3. Subgroup analysis (methodological considerations)

Figure S4. Adverse Events

**Table S1. Research question using PICOS structure**

| **Population** | Adults (18 years or older) diagnosed with osteoarthritis |
| --- | --- |
| **Intervention** | Traditional acupuncture |
| **Comparator(s)** | Usual care, no treatment, sham acupuncture |
| **Outcomes** | *Primary Outcome*   - Pain intensity   *Secondary Outcomes*   - Functional mobility - Health related quality of life   *Safety Outcomes*   - Adverse events |
| **Study design** | Prospective randomized controlled trials |

**Table S2. Study eligibility criteria**

| **Inclusion Criteria** | 1. Majority of studies (>80%) where participants are adults (18 years and older)) diagnosed with osteoarthritis.  2. Prospective, randomized controlled trials  3. Acupuncture administration. |
| --- | --- |
| **Exclusion Criteria** | 1. Studies involving animals.  2. All patients received some sort of acupuncture-based intervention  3. Quasi-randomized controlled trials (e.g., alternate randomization, randomization according to hospital number) and non-randomized trials (e.g. cohort and case-control studies).  4. Studies in which electro-acupuncture was the intervention, or electrical stimulation of the needles was performed. |

**Table S3. PubMed/MEDLINE search strategy**

| 1. Osteoarthritis [MeSH Terms] 2. Osteoarthritis OR Osteoarthritides OR osteoarthroses OR Degenerative arthritis OR Degenerative Arthritides OR Osteoarthrosis Deformans 3. Acupuncture[MeSH Terms] 4. Acupuncture OR Acupuncture therapy OR (Acupuncture AND therap*) 5. (randomized controlled trial[pt] OR controlled clinical trial[pt] OR randomized[tiab] OR placebo[tiab] OR drug therapy[sh] OR randomly[tiab] OR trial[tiab] OR groups[tiab] NOT (animals[mh] NOT humans [mh]) 6. (#1 OR #2) AND (#3 OR #4) AND #5 |
| --- |

**Table S4. Needle location for each trial**

| Scharf, 2006 | ST34; ST36; Xiyan; SP9; SP10; GB34 |
| --- | --- |
| Williamson, 2007 | ST35; ST36; Xiyan; SP9; SP10; GB34; Liv3 |
| Foster, 2007 | ST34; ST35; ST36; Xiyan; SP9; SP10; GB34 |
| Witt, 2006 | ST34; ST35; ST36; Xiyan; SP9; SP10; GB33; GB34 |
| White, 2011 | Not specified |
| Vas, 2004 | ST36; ST40; SP9; GB34; EXLE5 |
| Levi-Ari, 2011 | ST35; Xiyan; SP5; GB34 |
| Soni, 2012 | ST34; ST36; SP9; SP10; GB34; L3; L8 |
| Haslam; 2001 | ST44; GB29; GB30; GB34; GB43; L14 |
| Saleki, 2013 | ST34; ST35; ST36; SP9; SP10; GB34 |
| Ashraf, 2013 | ST35; ST36; ST44; GB34; EX31; EX32; DU 20; BL60 |
| Dickens | Not specified |

**Figure S1. Funnel plot for pain intensity**

SE = standard error; MD = mean difference

**Figure S2. Subgroup analysis**

**Mean Difference**

**IV, Random, 95% CI**

**Point**

**Estimate**

**Lower**

**limit**

**Upper**

**limit**

-0.38

-0.69

-0.06

-0.06

-0.56

0.44

-0.30

-0.65

0.05

-0.27

-0.67

0.14

-0.25

-0.57

0.07

**Duration of Intervention**

*More than 4 weeks*

*Four weeks or less*

**Type of Comparator**

*Sham acupuncture*

*Other comparator*

**Number of sessions**

*Ten or more*

*Less than 10*

-0.59

-1.18

-0.01

**-2.00**

**-1.00**

**0.00**

**1.00**

**2.00**

**Favours Acupuncture**

**Favours Control**

**Clinical considerations**

CI = Confidence interval; IV = inverse variance

**Figure S3. Subgroup analysis**

**Mean Difference**

**IV, Random, 95% CI**

**Point**

**Estimate**

**Lower**

**limit**

**Upper**

**limit**

-0.59

-1.18

-0.00

-0.21

-0.51

0.09

-0.22

-0.66

0.22

-0.33

-0.67

0.01

**Risk of bias**

*Low risk*

*Unclear/ High risk*

**Number of centres**

*Single centre trials*

*Multicentre trials*

**Methodological considerations**

**-2.00**

**-1.00**

**0.00**

**1.00**

**2.00**

**Favours Acupuncture**

**Favours Control**

CI = Confidence interval; IV = inverse variance

**Figure S4. Adverse Events**

Boxes and horizontal lines represent point estimates, varying in size according to the weight in the analysis, and 95% confidence intervals. Chi^2^ = Chi-squared df = degrees of freedom; CI = Confidence interval; I^2^ = I-squared; M-H = Mantel-Haenszel; P = P value; Tau^2^ = Tau-squared; Z = Z score
